# Neuroinflammation in Parkinson's Disease and Related Disorders: A Lesson from Genetically Manipulated Mouse Models of **α**-Synucleinopathies

**DOI:** 10.1155/2012/271732

**Published:** 2012-03-25

**Authors:** Kazunari Sekiyama, Shuei Sugama, Masayo Fujita, Akio Sekigawa, Yoshiki Takamatsu, Masaaki Waragai, Takato Takenouchi, Makoto Hashimoto

**Affiliations:** ^1^Division of Sensory and Motor Systems, Tokyo Metropolitan Institute of Medical Science, Tokyo 156-0057, Japan; ^2^Department of Physiology, Nippon Medical School, Tokyo 113-8602, Japan; ^3^Division of Animal Sciences, National Institute of Agrobiological Sciences, Tsukuba, Ibaraki 305-8634, Japan

## Abstract

Neuroinflammation in Parkinson's disease (PD) is a chronic process that is associated with alteration of glial cells, including astrocytes and microglia. However, the precise mechanisms remain obscure. To better understand neuroinflammation in PD, we focused on glial activation in *α*-synuclein (*α*S) transgenic and related model mice. In the majority of *α*S transgenic mice, astrogliosis was observed concomitantly with accumulation of *α*S during the early stage of neurodegeneration. However, microglia were not extensively activated unless the mice were treated with lipopolysaccharides or through further genetic modification of other molecules, including familial PD risk factors. Thus, the results in *α*S transgenic mice and related model mice are consistent with the idea that neuroinflammation in PD is a double-edged sword that is protective in the early stage of neurodegeneration but becomes detrimental with disease progression.

## 1. Introduction

The neurodegenerative brain in Parkinson's disease (PD) is characterized by protein aggregation of *α*-synuclein (*α*S), formation of Lewy bodies and Lewy neuritis, extensive loss of dopaminergic neurons, and gliosis in the substantia nigra [1, 2]. Similar *α*S pathologies have been observed in various types of *α*-synucleinopathies, including Dementia with Lewy Bodies (DLB), multiple system atrophy (MSA), neurodegeneration with brain iron accumulation, type 1, and the Lewy body variant of Alzheimer's disease (AD) [2]. Mechanistically, a wealth of data has suggested that neurotoxicity is well correlated with formation of oligomers and protofibrils of *α*S [3]. Immature fibrils of *α*S may be causative for diverse neurodegenerative alterations such as mitochondrial damage, increased endoplasmic reticulum stress, loss of membrane integrity, dysfunction of the ubiquitin-proteasome system, lysosomal leakage, and Golgi fragmentation [4]. In contrast, mature fibrils formed in the late stage of aggregation of *α*S might be protective since they may capture toxic and metastatic immature forms of *α*S, sequestering them into Lewy bodies [3].

In addition to cell-autonomous neurotoxicity due to aggregation of *α*S, mounting evidence from histology and cell biology has suggested that non-cell-autonomous neuroinflammation may be crucial for neurotoxicity since aberrant activation of glial cells may stimulate inflammation, leading to neuronal cell death [5, 6]. This view has been supported by recent progress in genetic studies. In particular, a genomewide association studies (GWAS) demonstrated association of a single nucleotide polymorphism (SNP) of leucine-rich repeat kinase 2 (LRRK2) (park8) with both sporadic PD [7, 8] and other inflammation-related disorders such as Crohn's disease [9] and leprosy [10], establishing a new concept that neuroinflammation may play a primary role in neurodegeneration in PD.

Despite the central role of neuroinflammation in the pathogenesis of PD and related *α*-synucleinopathies, the precise mechanism is still unclear. Indeed, many concepts of neuroinflammation have been based on the results of Parkinsonian models induced by treatment with agents such as 1-methyl-4-phenyl-1,2,3,6-tetrahydropyridine (MPTP) and 6-hydroxydopamine, in which microglia are activated within several hours after treatment and supply multiple neurotoxic factors, including tumor necrosis factor-*α*, nitric oxide, interleukin-1*β*, and reactive oxygen species, that drive progressive neurodegeneration [11]. However, the extent to which results obtained from such acute models are applicable to neuroinflammation in PD brains is yet to be determined, since neuroinflammation is a chronic phenomenon that occurs over decades and has a time course associated with alteration of different types of glial cells. Thus, the main objective of this paper is to explore the mechanism of neuroinflammation based on information derived from transgenic (tg) mouse models of *α*-synucleinopathies.

## 2. Role of Astroglia in Neuroinflammation in **α**-Synucleinopathies

Since the discovery of missense mutations of *α*S in familial cases of PD, many tg mouse models of *α*-synucleinopathies expressing full-length human wild-type or disease-linked mutant *α*S have been created using neuron-specific promoters such as platelet-derived growth factor-*β*  [12], Thy-1 [13, 14], and prion protein [15, 16]. These *α*S tg model mice recapitulate similar *α*S pathologies to those of PD brains, such as formation of *α*S-positive accumulations in neuronal cell bodies and neurites, a reduction of the density of tyrosine hydrolase-positive terminals in the striatum, and *α*S aggregation in detergent-insoluble fractions based on biochemical analyses, although behavioral disorders including deterioration of rotarod performance might be due to degeneration of cortical neurons associated with *α*S accumulation.

Reactive astrocytes are ubiquitously observed in various neurodegenerative disorders. Consistent with this, astrocytic gliosis has been shown in all *α*S tg mice. However, the mechanisms through which astrocytes are consistently activated in the brains of *α*S tg mice are elusive. One possible mechanism is direct stimulation of astrocytes by *α*S derived from degenerating neurons. In support of this view, recent data suggest that *α*S may be released from neurons via a nonclassical secretory pathway [17] and may therefore exert paracrine effects in the extracellular environment, which might be related to propagation of *α*S to adjacent cells, including neurons and astrocytes [18, 19]. Indeed, a sensitive enzyme-linked immunosorbent assay in combination with *in vivo* microdialysis was used to demonstrate the presence of relatively high concentrations of *α*S in brain interstitial fluids [20]. Furthermore, it was shown that *α*S-containing inclusion bodies are present in astrocytes [21], although it is still possible that astrocytes themselves produce *α*S.

Since astrocytes have a multitude of protective functions, including regulation of the ionic milieu in the intercellular space, uptake and/or breakdown of glutamate, and maintenance of the integrity of the blood-brain barrier [22], it is likely that, as long as astrocytes are intact in the early stage of neurodegeneration, they may be protective against neurodegeneration. Astrocytes may perceive the degenerative conditions of neurons through detection of neuron-derived *α*S and are subsequently activated to protect neurons. However, such reactive astrocytes are exposed to increasing toxicity of *α*S oligomers and/or protofibrils, until they are no longer protective (loss of function). Alternatively, astrocytes might become aberrantly activated during the long time course of neurodegeneration (gain of function). It is also possible that the presence of such aberrantly reactive astrocytes could be a prerequisite for activation of microglia. Whichever the case, astrocytes may be regarded as critical regulators of neuroinflammation. Notably, this notion is supported by recent work by Gu and colleagues, who created tg mice with astrocytic overexpression of A53T mutant *α*S under regulation by the tetracycline operator [23]. These mice exhibited extensive phenotypes, such as rapid progressive paralysis, accumulation of *α*S aggregates, expansion of reactive astrogliosis, and microglial activation, whereas the normal function of astrocytes seemed to be compromised, as evidenced by cerebral microhemorrhage and downregulation of astrocytic glutamate transporters.

## 3. Role of Environmental Factors in Neuroinflammation in **α**-Synucleinopathies

Compared to the extensive formation of reactive astrocytes, activated microglia are rarely detected in *α*S tg mice. The mechanism is still elusive, but it is possible that additional factors might be required to enhance neuroinflammation associated with microglia in these mice. Indeed, the loss of nigral dopaminergic neurons in idiopathic PD is believed to result from interactions between genetic susceptibility and environmental factors [24]. Based on this idea, Gao and colleagues injected an inflammagen, lipopolysaccharide (LPS), into the substantia nigra of A53T mutant *α*S tg mice and found that neuroinflammation was associated with dopaminergic neuronal death and accumulation of insoluble aggregated *α*S as cytoplasmic inclusions in nigral neurons [25]. Furthermore, nitrated/oxidized *α*S was detected in these inclusions and inhibition of microglia-derived nitric oxide and superoxide resulted in neuroprotection in neuron-glia cultures, suggesting that nitric oxide and superoxide released by activated microglia may be mediators that link inflammation and abnormal *α*S in mechanisms of PD neurodegeneration. Essentially similar results were obtained by intraperitoneal injection of LPS in *α*S tg mice [26]. These results led the authors to propose a two-hit model in which mutant *α*S and inflammation work in concert to mediate chronic PD neurodegeneration [11]. Supporting the role of viral infections in the pathogenesis of *α*-synucleinopathies, it was shown that intranasal administration of the neurotropic virus H5N1 resulted in *α*S aggregation and microglial activation [27]. Since it is still unclear whether other types of infections are related to the elevations of PD risks [11], further epidemiologic studies are warranted to test this intriguing hypothesis. 

## 4. Alteration of Familial PD Risk Factors Leads to Enhanced Neuroinflammation

Besides *α*S (PARK1, PARK4), there is increasing evidence to suggest that familial PD risk factors may be involved in both cell-autonomous and non-cell-autonomous neurotoxicities. The results of knockout mouse studies of autosomal recessive factors such as parkin (PARK2), PINK1 (PARK6), and DJ-1 (PARK7) suggest that, in addition to cell-autonomous protective functions for neurons, these factors may be involved in the negative regulation of neuroinflammation. Parkin, mutations of which are present in at least 50% of patients with autosomal recessive juvenile parkinsonism [29], is a ubiquitin E3 ligase [30]. This finding led to establishment of the importance of the ubiquitin/proteasome system in *α*-synucleinopathies. Subsequently, PINK1, a mitochondrially targeted Ser/Thr kinase of which mutations are the second most frequent cause of autosomal recessive young-onset PD [31], was shown to cooperate with parkin in maintenance of mitochondrial quality, and mutations in these genes were causative for mitophagy [32]. Although Parkin (−/−) mice do not display nigrostriatal pathway degeneration, these mice displayed subtle fine-motor deficits and selective loss of DA neurons in the substantia nigra when intraperitoneally treated with LPS [33]. Similarly, the numbers of dopaminergic neurons and levels of striatal DA and DA receptors were unchanged in PINK1(−/−) mice; however, these mice had increased levels of IL-1*β*, IL-12, and IL-10 in the striatum after peripheral challenge with LPS, and PINK1(−/−) embryonic fibroblasts showed decreased basal and inflammatory cytokine-induced nuclear factor kappa-*β* activity [34]. Thus, Parkin and PINK1 deficiencies collectively increase the vulnerability of nigral DA neurons to inflammation-related degeneration.

Loss of function of DJ-1 has been linked to autosomal recessive PD [35] and Parkinsonism-dementia-amyotrophic lateral sclerosis complex [36]. Since DJ-1 is abundantly expressed in reactive astrocytes [37], it was assumed that DJ-1 might be involved in regulation of astrocytic activation or some astrocytic functions. In this context, Waak and colleagues showed that LPS treatment of astrocyte cultures from DJ-1(−/−) mice displayed enhanced features of inflammation, such as nitric oxide, inducible nitric oxide synthase, cyclooxygenase-2, and IL-6, compared with LPS-treated astrocytes from littermate controls [38]. These results suggest that DJ-1 might act as a negative regulator of proinflammatory responses in astrocytes and that loss of DJ-1 might contribute to PD pathogenesis through deregulation of astrocytic neuroinflammatory damage [38].

Compared to autosomal recessive factors, it is more likely that the autosomal dominant factor, LRRK2, may play a critical role in the pathogenesis of sporadic PD. Indeed, mutations in LRRK2 are linked to the most common familial autosomal dominant types of late-onset PD, as well as some cases of sporadic PD [39, 40]. Notably, many LRRK2 mutation carriers exhibit typical PD symptoms that are clinically indistinguishable from sporadic PD [40]. Consistent with this, findings from GWAS recently demonstrated that two autosomal dominant genes for familial PD, *α*S and LRRK2, are strongly associated with sporadic PD [7, 8]. In this context, *α*S and LRRK2 appear to be commonly involved in pathologies such as impairment of cytoskeleton dynamics, dysregulation of the protein degradation system, and enhanced protein aggregation. Thus, it was predicted that, similarly to *α*S, LRRK2 might be involved in neuroinflammation in sporadic cases of PD and related *α*-synucleinopathies.

The role of LRRK2 in neuroinflammation was demonstrated by Lin and colleagues through generation of LRRK2 knockout (−/−) and transgenic mice expressing human wildtype, G2019S mutant, or kinase-domain-deletion LRRK2 under the transcriptional control of a tetracycline operator [41]. Neither deletion nor overexpression of LRRK2 caused overt gross neuropathological abnormalities in mutant mice. However, cross-experiments showed that the presence of excess LRRK2 greatly accelerated the progression of neuropathological abnormalities developed in PD-related A53T *α*S transgenic mice, such as abnormal aggregation and somatic accumulation of *α*S impairment of microtubule dynamics, Golgi organization, and the ubiquitin-proteasome pathway. In these bigenic mice, the neurodegeneration was further characterized by neuronal cell death associated with reactive astrocytes and activated microglia. Conversely, inhibition of LRRK2 expression reduced the aggregation/accumulation of *α*S and delayed progression of *α*S-mediated neuropathology. These results suggest that LRRK2 might be involved in the toxic gain of functions of *α*S. In this regard, it is worth noting that LRRK2-knockout mice showed PD-like pathology, such as *α*S aggregation and impairment of autophagy-lysosome pathway in their kidneys [42]. Thus, the pathogenic mechanisms of LRRK2 are complicated and one possible interpretation may be the dominant negative mechanism of LRRK2 for the potential role of this molecule in protein degradation. 

Curiously, GWAS findings have demonstrated that a certain SNP in LRRK2 is associated with inflammatory bowel diseases such as Crohn's disease and ulcerative colitis [9]. Furthermore, SNPs of LRRK2 and parkin were both associated with leprosy, a chronic infectious disease caused by *Mycobacterium leprae *[10]. Thus, PD and *α*-synucleinopathies are now genetically considered to be within the spectrum of inflammatory diseases in which LRRK2 and parkin might be involved. However, since the SNPs of LRRK2 and Parkin that are positively linked to inflammatory bowel diseases and leprosy differ from those linked to PD [7, 8], it is unclear whether the molecular mechanisms are similar in *α*-synucleinopathies and other inflammation-related disorders.

## 5. Analysis of Neuroinflammation in DLB-Linked P123H**β**-Syn tg Mice

Although previous studies have shown that *β*S plays a neuroprotective role [43, 44], evidences are accumulating to suggest that alteration of this molecule may also stimulate neurodegeneration. In support of this notion, *β*S and *γ*-synuclein (*γ*S) are both associated with neuritic pathology, such as in dystrophic neurites and spheroid structures, in the brains of sporadic cases of PD, DLB [45], and neurodegeneration with brain iron accumulation, type I [46]. Thus, it is probable that not only *α*S but also other synuclein family members [47] might be involved in neuroinflammation.Two missense mutations of *β*S have been discovered in unrelated DLB [48]: a valine to methionine substitution at position 70 (V70M) was found in a sporadic DLB case in Japan, while a proline to histidine mutation (P123H) was identified in familial DLB cases in Seattle. Biochemical analysis *in vitro* and cell culture studies suggested that nonamyloidogenic *β*S was converted to aggregate-prone protein through gene mutations, contributing to the pathogenesis of familial DLB [49, 50].

**Figure 1:**
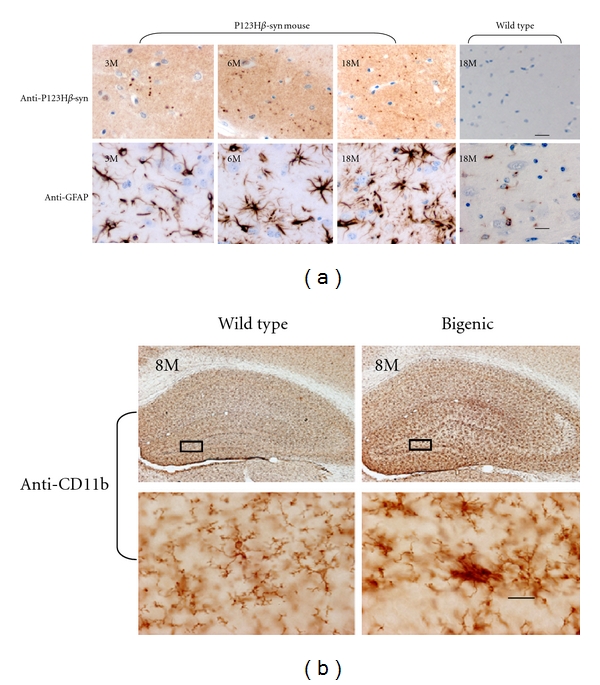
Characterization of glial activation in a P123H*β*S tg mouse and an *α*S/P123H*β*S bigenic mouse.(a) Concomitant with P123H*β*S-positive dot-like axonal accumulation (upper), GFAP-positive reactive astrocytes were observed in the hippocampus of a P123H*β*S tg mouse, but not in a wild-type mouse (lower). Scale bar = 20 *μ*m. (b) Immunostaining with anti-CD11b antibody showed microglial activation in an *α*S/P123H*β*S bigenic mouse, but not in a wild-type mouse. Scale bar = 20 *μ*m. The figures in (a) and (b) were reprinted from Fujita et al., [28], with permission, or are unpublished data related to the same paper.

To assess the consequences of excess expression of P123H*β*S, we generated tg mice overexpressing P123H*β*S under control of the Thy-1 promoter [28]. These mice were characterized by memory dysfunction at a relatively early age (~6 mo). Histopathological analyses revealed extensive neuritic pathology that started at the same stage. P123H*β*S accumulated in various brain regions, including apical dendrites in the cortex and axonal deposits in the hippocampus (Figure 1(a)). Notably, the same regions were accompanied by massive gliosis, as revealed by an increased level of glial fibrillary-acidic-protein-(GFAP-) positive astroglia (Figure 1(a)). In contrast to P123H*β*S tg mice, neither abnormal immunostaining of P123H*β*S nor gliosis was observed in non-tg littermates or in mice overexpressing wild-type *β*S [28]. Extensive P123H*β*S-immunoreactive axonal swellings were formed in the striatum and globus pallidus in the late stage, but neither neuronal cell death nor microglia activation was observed. 

Cross-breeding of a P123H*β*S tg mouse with an *α*S tg mouse greatly enhanced neurodegeneration phenotypes, which is reminiscent of the synergistic effects between mutant *β*S and *α*S *in vitro* [28]. In addition to aggregation of P123H*β*S and *α*S, tyrosine hydroxylase was decreased and motor dysfunctions were observed. Furthermore, microglia were extensively activated (Figure 1(b)) concomitantly with dark cell neurons, one type of neuronal cell death. These results suggest that P123H*β*S may contribute to the *α*S pathology in mouse brain.

Regarding *γ*S, Ninkina and colleagues generated transgenic mice expressing high levels of mouse *γ*S under control of the Thy-1 promoter [51]. These mice developed severe age and transgene dose-dependent neuropathology and motor deficits and died prematurely. Histopathological changes included aggregation and accumulation of *γ*S in neuronal cell bodies and processes, in addition to the presence of *γ*S-positive spheroids and dystrophic neuritis. Astrogliosis was observed, but activation of microglia was not described, suggesting that neuroinflammation was not extensive in these mice

## 6. Role of Oligodendrocytes in Neuroinflammation in **α**-Synucleinopathies

It is generally thought that oligodendrocytes are not involved in neuroinflammation in PD. Nonetheless, the results obtained from *α*S tg mice in a multiple system atrophy (MSA) model may be important for understanding the role of glial cells in neuroinflammation in *α*-synucleinopathies [52–54]. Since MSA is histologically characterized by *α*S-immunoreactive cytoplasmic inclusions in oligodendrocytes [2], the pathogenic mechanism of MSA may be attributable to alteration of oligodendrocytes by accumulation of *α*S. In this context, tg MSA mouse models have been established with expression of full-length wild-type or disease-linked mutant *α*S under control of oligodendroglia-specific promoters such as 2′,3′-cyclic nucleotide 3′-phosphodiesterase [52], myelin basic protein [53], and proteolipid protein [54]. These *α*S tg mice develop extensive *α*S-immunoreactive inclusions in oligodendrocytes in various brain regions, including the neocortex, basal ganglia, cerebellum, and brainstem, which are accompanied by myelin and neuronal damage and motor deficits, recapitulating features of MSA [52–54].

There are at least two mechanisms through which altered oligodendrocytes may stimulate neurodegeneration. First, oligodendrocytes are specifically involved in myelination [55], and their alterations may dysregulate myelination and ultimately lead to axonal degeneration and synaptic loss. Alternatively, since oligodendrocytes are the predominant cells for glutamate clearance in human white matter [56], alteration of oligodendrocytes might underlie accumulation of high extracellular glutamate and produce an increased risk for glutamate excitotoxicity.

Astrogliosis was observed in all reported MSA mice, but no microglial activation was described [52–54]. In this regard, it is worth noting that microglia may play a protective role against oligodendroglial alteration and neurodegeneration [57]. Ablation of toll-like receptor 4 (TLR-4) in a tg mouse model of MSA with oligodendroglial *α*S overexpression augmented motor disability and enhanced loss of nigrostriatal dopaminergic neurons, which were associated with increased brain levels of *α*S linked to disturbed TLR-4-mediated microglial phagocytosis of *α*S [57]. Taken together, these results suggest that both astrocytes and microglia have protective roles in *α*S tg mice in the MSA model.

## 7. Conclusions and Perspectives

**Figure 2:**
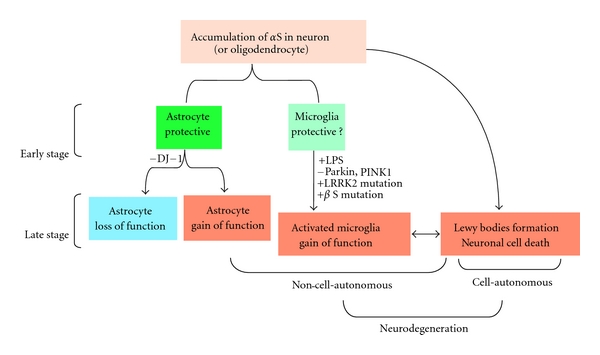
Schematic hypothesis of glial activations in the *α*S tg mouse. Astrocytes may quickly respond to *α*S released from degenerating neurons to protect against neurodegeneration. During the long time course of neurodegeneration, these activated astrocytes may lose their protective properties or might be aberrantly activated, leading to stimulation of neuroinflammation. This process may be stimulated by loss of function of DJ-1. In contrast, microglia may be protective during the early stage of neurodegeneration and are not readily activated unless they are stimulated with LPS or with alterations of other familial PD risk factors such as Parkin, PINK1, LRRK2, and other members of the synuclein family of peptides, including mutant *β*S.

The results from genetically engineered mouse models of PD and related *α*-synucleinopathies strongly suggest that the mechanism of glial activation of chronic model *α*-synucleinopathies in *α*S tg mice is distinct from that in drug (e.g., MPTP)-induced acute parkinsonian models, which are characterized by rapid and extensive activation of microglia upon drug treatment, followed by mild activation of astrocytes [11]. In *α*S tg mice, astrocytes may respond to *α*S released from degenerating neurons to protect against neurodegeneration (Figure 2). During the long time course of neurodegeneration, these activated astrocytes may lose their protective properties or might be aberrantly activated, leading to stimulation of neuroinflammation. In contrast, microglia are not readily activated unless they are stimulated with an inflammagen such as LPS or through alterations of other familial PD risk factors such as parkin, PINK1 and LRRK2 (Figure 2). The finding that microglia are not easily activated in *α*S tg mice raises the possibility that activation of microglia might be a relatively late event during chronic development of *α*-synucleinopathies. Thus, this may be comparable to Lewy body formation and massive neuronal cell death, both of which are histopathological hallmarks of autopsy brains. In support of this notion, we found that microglia are activated (Figure 1(b)) concomitantly with neuronal cell death in a bigenic mouse expressing both *α*S and P123H*β*S, but not in single tg mouse expressing *α*S or P123H*β*S (Figures 1 and 2) [28]. It is also possible that, similarly to astrocytes, microglia are protective during the early stage of neurodegeneration. This possibility may be supported by a crossing experiments with *α*S tg MSA model mice and TLR-4 knockout mice [57].

It is unclear whether *α*S tg mice are appropriate for analysis of the possible alteration of astrocytes and microglial activation in the late stage of PD. However, *α*S tg mice may provide a means to address fundamental aspects of the early stage of *α*-synucleinopathies in the period in which astrocytes and microglia have protective roles against neurodegeneration. An understanding of the pathogenic mechanism in the early stage of a disease is essential for early diagnosis and implementation of a protective strategy. *α*S tg mice models are invaluable for this purpose, including evaluation of the early stage of neuroinflammation.

## References

[1] Hashimoto M, Masliah E (1999). *α*-Synuclein in Lewy body disease and Alzheimer’s disease. *Brain Pathology*.

[2] Trojanowski JQ, Goedert M, Iwatsubo T, Lee VMY (1998). Fatal attractions: abnormal protein aggregation and neuron death in Parkinson’s disease and Lewy body dementia. *Cell Death and Differentiation*.

[3] Rochet JC, Lansbury PT (2000). Amyloid fibrillogenesis: themes and variations. *Current Opinion in Structural Biology*.

[4] Beyer K, Domingo-Sabat M, Ariza A (2009). Molecular pathology of lewy body diseases. *International Journal of Molecular Sciences*.

[5] Block ML, Zecca L, Hong JS (2007). Microglia-mediated neurotoxicity: uncovering the molecular mechanisms. *Nature Reviews Neuroscience*.

[6] Kim YS, Joh TH (2006). Microglia, major player in the brain inflammation: their roles in the pathogenesis of Parkinson’s disease. *Experimental and Molecular Medicine*.

[7] Satake W, Nakabayashi Y, Mizuta I (2009). Genome-wide association study identifies common variants at four loci as genetic risk factors for Parkinson’s disease. *Nature Genetics*.

[8] Simon-Sanchez J, Schulte C, Bras JM (2009). Genome-wide association study reveals genetic risk underlying Parkinson’s disease. *Nature Genetics*.

[9] van Limbergen J, Wilson DC, Satsangi J (2009). The genetics of Crohn’s disease. *Annual Review of Genomics and Human Genetics*.

[10] Zhang FR, Huang W, Chen SM (2009). Genomewide association study of leprosy. *New England Journal of Medicine*.

[11] Liu B, Gao HM, Hong JS (2003). Parkinson’s disease and exposure to infectious agents and pesticides and the occurrence of brain injuries: role of neuroinflammation. *Environmental Health Perspectives*.

[12] Masliah E, Rockenstein E, Veinbergs I (2000). Dopaminergic loss and inclusion body formation in *α*-synuclein mice: implications for neurodegenerative disorders. *Science*.

[13] van der Putten H, Wiederhold KH, Probst A (2000). Neuropathology in mice expressing human *α*-synuclein. *The Journal of Neuroscience*.

[14] Rockenstein E, Mallory M, Hashimoto M (2002). Differential neuropathological alterations in transgenic mice expressing *α*-synuclein from the platelet-derived growth factor and Thy-1 promoters. *Journal of Neuroscience Research*.

[15] Giasson BI, Duda JE, Quinn SM, Zhang B, Trojanowski JQ, Lee VMY (2002). Neuronal *α*-synucleinopathy with severe movement disorder in mice expressing A53T human *α*-synuclein. *Neuron*.

[16] Lee MK, Stirling W, Xu Y (2002). Human *α*-synuclein-harboring familial Parkinson’s disease-linked Ala-53 → Thr mutation causes neurodegenerative disease with *α*-synuclein aggregation in transgenic mice. *Proceedings of the National Academy of Sciences of the United States of America*.

[17] Jang A, Lee HJ, Suk JE, Jung JW, Kim KP, Lee SJ (2010). Non-classical exocytosis of *α*-synuclein is sensitive to folding states and promoted under stress conditions. *Journal of Neurochemistry*.

[18] Desplats P, Lee HJ, Bae EJ (2009). Inclusion formation and neuronal cell death through neuron-to-neuron transmission of *α*-synuclein. *Proceedings of the National Academy of Sciences of the United States of America*.

[19] Lee HJ, Suk JE, Patrick C (2010). Direct transfer of *α*-synuclein from neuron to astroglia causes inflammatory responses in synucleinopathies. *The Journal of Biological Chemistry*.

[20] Emmanouilidou E, Elenis D, Papasilekas T (2011). Assessment of *α*-synuclein secretion in mouse and human brain parenchyma. *PLoS One*.

[21] Wakabayashi K, Hayashi S, Yoshimoto M, Kudo H, Takahashi H (2000). NACP/*α*-synuclein-positive filamentous inclusions in astrocytes and oligodendrocytes of Parkinson’s disease brains. *Acta Neuropathologica*.

[22] Benarroch EE (2005). Neuron-astrocyte interactions: partnership for normal function and disease in the central nervous system. *Mayo Clinic Proceedings*.

[23] Gu XL, Long CX, Sun L, Xie C, Lin X, Cai H (2010). Astrocytic expression of Parkinson’s disease-related A53T *α*-synuclein causes neurodegeneration in mice. *Molecular Brain*.

[24] Semchuk KM, Love EJ, Lee RG (1993). Parkinson’s disease: a test of the multifactorial etiologic hypothesis. *Neurology*.

[25] Gao HM, Kotzbauer PT, Uryu K, Leight S, Trojanowski JQ, Lee VMY (2008). Neuroinflammation and oxidation/nitration of *α*-synuclein linked to dopaminergic neurodegeneration. *The Journal of Neuroscience*.

[26] Gao HM, Zhang F, Zhou H, Kam W, Wilson B, Hong JS (2011). Neuroinflammation and *α*-synuclein dysfunction potentiate each other, driving chronic progression of neurodegeneration in a mouse model of Parkinson’s disease. *Environmental Health Perspectives*.

[27] Jang H, Boltz D, Sturm-Ramirez K (2009). Highly pathogenic H5N1 influenza virus can enter the central nervous system and induce neuroinflammation and neurodegeneration. *Proceedings of the National Academy of Sciences of the United States of America*.

[28] Fujita M, Sugama S, Sekiyama K (2010). A *β*-synuclein mutation linked to dementia produces neurodegeneration when expressed in mouse brain. *Nature Communications*.

[29] Kitada T, Asakawa S, Hattori N (1998). Mutations in the parkin gene cause autosomal recessive juvenile parkinsonism. *Nature*.

[30] Shimura H, Hattori N, Kubo SI (2000). Familial Parkinson disease gene product, parkin, is a ubiquitin-protein ligase. *Nature Genetics*.

[31] Valente EM, Abou-Sleiman PM, Caputo V (2004). Hereditary early-onset Parkinson’s disease caused by mutations in *PINK1*. *Science*.

[32] Youle RJ, Narendra DP (2011). Mechanisms of mitophagy. *Nature Reviews Molecular Cell Biology*.

[33] Frank-Cannon TC, Tran T, Ruhn KA (2008). Parkin deficiency increases vulnerability to inflammation-related nigral degeneration. *The Journal of Neuroscience*.

[34] Akundi RS, Huang Z, Eason J (2011). Increased mitochondrial calcium sensitivity and abnormal expression of innate immunity genes precede dopaminergic defects in *PINK1*-deficient mice. *PLoS One*.

[35] Bonifati V, Rizzu P, van Baren MJ (2003). Mutations in the *DJ-1* gene associated with autosomal recessive early-onset parkinsonism. *Science*.

[36] Annesi G, Savettieri G, Pugliese P (2005). *DJ-1* mutations and parkinsonism-dementia-amyotrophic lateral sclerosis complex. *Annals of Neurology*.

[37] Bandopadhyay R, Kingsbury AE, Cookson MR (2004). The expression of*DJ-1* (*PARK7*) in normal human CNS and idiopathic Parkinson’s disease. *Brain*.

[38] Waak J, Weber SS, Waldenmaier A (2009). Regulation of astrocyte inflammatory responses by the Parkinson’s disease-associated gene *DJ-1*. *The FASEB Journal*.

[39] Paisan-Ruiz C, Jain S, Evans EW (2004). Cloning of the gene containing mutations that cause *PARK8*-linked Parkinson’s disease. *Neuron*.

[40] Zimprich A, Biskup S, Leitner P (2004). Mutations in *LRRK2* cause autosomal-dominant parkinsonism with pleomorphic pathology. *Neuron*.

[41] Lin X, Parisiadou L, Gu XL (2009). Leucine-rich repeat kinase 2 regulates the progression of neuropathology induced by Parkinson’s-disease-related mutant *α*-synuclein. *Neuron*.

[42] Tong Y, Pisani A, Martella G (2009). R1441C mutation in *LRRK2* impairs dopaminergic neurotransmission in mice. *Proceedings of the National Academy of Sciences of the United States of America*.

[43] Hashimoto M, Rockenstein E, Mante M, Mallory M, Masliah E (2001). *β*-synuclein inhibits *α*-synuclein aggregation: a possible role as an anti-Parkinsonian factor. *Neuron*.

[44] Fan Y, Limprasert P, Murray IVJ (2006). *β*-synuclein modulates *α*-synuclein neurotoxicity by reducing *α*-synuclein protein expression. *Human Molecular Genetics*.

[45] Galvin JE, Uryu K, Lee VMY, Trojanowski JQ (1999). Axon pathology in Parkinson’s disease and Lewy body dementia hippocampus contains *α*-, *β*-, and *γ*-synuclein. *Proceedings of the National Academy of Sciences of the United States of America*.

[46] Galvin JE, Giasson B, Hurtig HI, Lee VMY, Trojanowski JQ (2000). Neurodegeneration with brain iron accumulation, type 1 is characterized by *α*-, *β*-, and *γ*-synuclein neuropathology. *American Journal of Pathology*.

[47] Clayton DF, George JM (1998). The synucleins: a family of proteins involved in synaptic function, plasticity, neurodegeneration and disease. *Trends in Neurosciences*.

[48] Ohtake H, Limprasert P, Fan Y (2004). *β*-synuclein gene alterations in dementia with Lewy bodies. *Neurology*.

[49] Wei J, Fujita M, Nakai M (2007). Enhanced lysosomal pathology caused by *β*-synuclein mutants linked to dementia with Lewy bodies. *The Journal of Biological Chemistry*.

[50] Wei J, Fujita M, Nakai M (2009). Protective role of endogenous gangliosides for lysosomal pathology in a cellular model of synucleinopathies. *American Journal of Pathology*.

[51] Ninkina N, Peters O, Millership S, Salem H, van der Putten H, Buchman VL (2009). *γ*-Synucleinopathy: neurodegeneration associated with overexpression of the mouse protein. *Human Molecular Genetics*.

[52] Yazawa I, Giasson BI, Sasaki R (2005). Mouse model of multiple system atrophy *α*-synuclein expression in oligodendrocytes causes glial and neuronal degeneration. *Neuron*.

[53] Shults CW, Rockenstein E, Crews L (2005). Neurological and neurodegenerative alterations in a transgenic mouse model expressing human *α*-synuclein under oligodendrocyte promoter: implications for multiple system atrophy. *The Journal of Neuroscience*.

[54] Kahle PJ, Neumann M, Ozmen L (2002). Hyperphosphorylation and insolubility of *α*-synuclein in transgenic mouse oligodendrocytes. *EMBO Reports*.

[55] Simons M, Trajkovic K (2006). Neuron-glia communication in the control of oligodencrocyte function and myelin biogenesis. *Journal of Cell Science*.

[56] deSilva TM, Kabakov AY, Goldhoff PE, Volpe JJ, Rosenberg PA (2009). Regulation of glutamate transport in developing rat oligodendrocytes. *The Journal of Neuroscience*.

[57] Stefanova N, Fellner L, Reindl M, Masliah E, Poewe W, Wenning GK (2011). Toll-like receptor 4 promotes *α*-synuclein clearance and survival of nigral dopaminergic neurons. *American Journal of Pathology*.

